# Short timescale wetting and penetration on porous sheets measured with ultrasound, direct absorption and contact angle†

**DOI:** 10.1039/c8ra01434e

**Published:** 2018-04-04

**Authors:** Krainer Sarah, Hirn Ulrich

**Affiliations:** Institute of Paper, Pulp and Fiber Technology, TU Graz Inffeldgasse 23 8010 Graz Austria ulrich.hirn@tugraz.at; CD Laboratory for Fiber Swelling and Paper Performance Inffeldgasse 23 8010 Graz Austria

## Abstract

In this study the short timescale penetration and spreading of liquids on porous sheets is investigated. Three measurement techniques are evaluated: ultrasonic liquid penetration measurement (ULP), contact angle measurement (CA) and scanning absorptiometry (SA). With each of these techniques liquid penetration as well as surface wetting can be measured. A quantitative comparison between the methods is carried out. For our studies we are using model liquids with tuneable surface tension, viscosity and surface energy which are the governing parameters for pore flow according to the Lucas–Washburn equation. Scanning absorptiometry turns out to be an adequate tool for direct measurement for liquid penetration. Ultrasonic liquid penetration showed a stable correlation (*R*^2^ = 0.70) to SA and thus also gives a suitable indication on the liquid penetration behaviour. Absorption of individual microliter drops measured in the CA instrument showed different results than the other two measurements. For characterisation of the wetting behaviour the measurement techniques gave substantially different results. We thus conclude that ULP and SA do not capture the wetting behaviour of liquids on paper in the same way as conventional contact angle measurement, it is unclear if their results are meaningful. Finally we are proposing two parameters indicating a combination of liquid penetration and wetting, the slope of the contact angle over time d*θ*/d*t* and a contact angle calculated from SA. These two parameters are moderately correlated, supporting the idea that they are indeed capturing a combination of liquid penetration and wetting. While our investigations are restricted to paper, we believe that the methods investigated here are generally applicable to study liquid absorption in thin porous media like microfluidic paper based analytical devices, thin porous storage media, membranes and the like. Our findings are highlighting the importance to have a match in timescale (time for penetration and wetting) and size scale (liquid amount supplied) between the testing method and the actual use case of the material, when analyzing wetting and penetration on porous materials.

## Abstract 2

Liquid penetration and wetting on thin, porous media is studied using three different measurement methods, and using testing liquids with tailored viscosity, polarity and surface tension.

## Graphical abstract

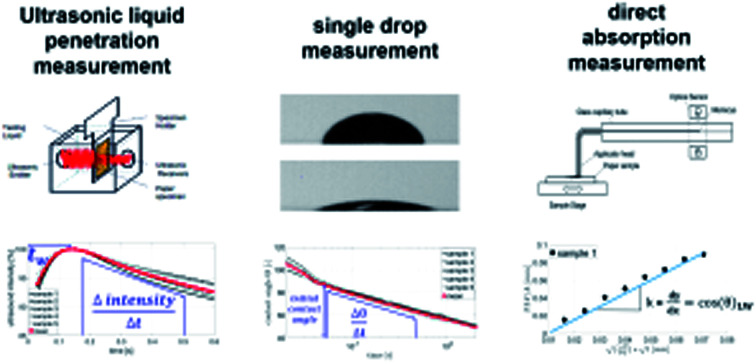

## 1 Introduction

Wetting and liquid absorption in thin, porous materials is a relevant performance characteristic *e.g.* for microfluidic paper based analytical devices, thin porous storage media or membranes. Also High Speed Inkjet (HSI) printing is influenced by the penetration behaviour of the fluid into the printing substrate, often paper.^1,2^ There are three main challenges studying liquid penetration and spreading in paper and other thin, porous media. First the thickness of the material is low, typically around 100 μm. Second paper has a high in-plane inhomogeneity which necessitates testing of either a large sample area or measurement of many specimen sampled over a large specimen area.

Finally liquid penetration and spreading in HSI printing are taking place within a few hundred milliseconds, requiring measurements with high time resolution. This has lead to the application of various analytical approaches. Commonly used techniques in paper and printing industry are ultrasound measurement (ultrasonic liquid penetration – ULP), scanning absorptiometry (SA) and contact angle measurement (CA). The application of these techniques in characterization of penetration speed and wetting was subject of several studies.^2–12^ In our study we are using these instruments for both, measurement of liquid penetration and for surface wetting.

The common method to determine the wetting behaviour of liquids is the contact angle measurement (CA). The contact angle of a drop, placed on the surface of a substrate, is filmed and measured from the images. The change of contact angle over time is influenced by the spreading of the drop and by the penetration of the liquid into the paper.^13–16^ Ultrasonic liquid penetration (ULP) is delivering a measure for surface wetting by measuring the time between liquid contact and the highest signal intensity. Also scanning absorptiometry (SA) can provide information on the wetting behaviour. The wetting parameter here is a calculated contact angle, computed from the measured liquid penetration using the Lucas–Washburn equation.^17–19^

For liquid penetration ultrasonic measurement is available in different configurations. All of them indicate the liquid penetration into paper and the wetting behaviour using ultrasound intensity.^3,7–9,20–22^ For example Sharma^8^ showed a correlation of ULP and inkjet print quality parameters for photographic papers. Liquid penetration is also measured using scanning absorptiometry. SA evaluates the liquid absorption per unit area at a specific contact time, it is a direct measure for the penetration speed. The potential of the SA for characterising the direct liquid uptake has been studied and shows good results for measuring penetration.^6,12,17^ Liquid penetration of single droplets has been investigated in this study using the contact angle instrument. The change in drop volume over time is calculated from an image sequence taken by the CA instrument.

### 1.1 Aim of the work

In this study we are comparing measurement of liquid penetration and surface wetting using ultrasonic liquid penetration measurement (ULP), contact angle measurement (CA) and scanning absorptiometry (SA). The techniques are investigated for their potential to measure penetration and wetting at different time scales, depending on the liquid (*e.g.* approximately 200 ms for fast penetration and 1.5 s for slow penetration). We are reporting results for liquid penetration in paper, however the findings should also be relevant for liquid absorption and wetting into other thin, porous materials.

For testing we are using 4 HSI inks and 5 water based model liquids with defined surface tension, viscosity and polarity in terms of Hansen solubility parameters. As discussed in Section 2.6 these three parameters are the governing features for liquid capillary penetration. The model liquids have been designed for decoupled tuning of these key liquid characteristics. The testing liquids are applied to four different papers with different degrees of liquid absorption and spreading. The chosen combination of papers and liquids are representative for the spectrum of the materials in the high speed inkjet printing process.

A quantitative comparison of the results from the different test outcomes is carried out in terms of linear regression and the suitability of the three methods to measure liquid penetration and wetting behavior is evaluated. The influence of time scale and size scale of penetration and wetting will be discussed with respect to the different measurements techniques and their results.

## 2 Materials and methods

All measurements have been conducted in a climate room under defined temperature (23 °C) and humidity (50% relative humidity) according to ISO 187:1990. The papers were then stored in the climate room for at least 24 hours to ensure they have reached equilibrium moisture content, as indicated in ISO standard 187:1990.

### 2.1 Papers

The paper grade significantly influences the penetration process. Therefore we investigated the performance of all liquids on four different wood free fine papers from an industrial supplier. These are an unsized HSI treated paper grade (DNS High Speed Inkjet CF by Mondi), an HSI unsized, pigmented paper (NEUJET Matte by Mondi) and an AKD sized paper (IQ ALLROUND by Mondi). The fourth paper, the unsized and untreated grade, is basically the unsized HSI paper grade (DNS High Speed Inkjet CF) without the surface treatment. The paper types are differing in terms of sizing (hydrophobisation) and surface treatment, covering the commercially available papers for office- and high speed inkjet printing papers.

The papers were characterized in terms of composition and pore structure, see Table 1. All of them are made of industrial bleached hardwood pulp. The common method for determination of grammage is the EN ISO 536. Filler content refers to the amount of CaCO_3_ filler particles in the paper. The filler is a commercial PCC (precipitated calcium carbonate) grade, filler content is measured according to DIN 54370. The pigmented paper grade has a low grammage surface sizing, about 3 g m^−2^ per side, consisting of a mixture of starch and clay. HSI surface treatment is a surface application of CaCl_2_ to trigger precipitation of the ink pigments on the paper surface and reduce penetration of the pigments into the bulk of the paper. The porosity and the pore diameter was obtained from mercury intrusion porosimetry, a standard method to characterize microscale pore size distributions.^23–26^ We utilized an Autopore IV 9500 from Micromeritics Instrument Corp.^27,28^

**Table 1:** Properties of the papers used: grammage, filler content, surface pigmentation, HSI-surface treatment, and porosity

Properties	AKD sized	Unsized	Pigmented	Unsized & untreated
Grammage [g m^−2^]	77.2	78.5	79.89	97.2
Filler content [%]	13.52	21.51	22.98	21.51
Pigmentation [g m^−2^]	0	0	4	0
HSI surface treatment	No	Yes	Yes	No
Porosity [%]	20.6	38.8	23.6	40.3
Avg. pore diameter [μm]	4.9	2.6	3.2	3.9

### 2.2 Testing liquids

Five water based model liquids have been prepared with respect to their viscosity, surface tension and polarity. The polarity is defined through three Hanson solubility parameters.^29^ The parameters are coordinates in a three dimensional coordinate system, the HSP space. d*D* describes the dispersion forces between molecules, d*P* the dipolar forces and d*H* the forces from hydrogen bonds. The tested model liquids are DI water, 80% water/20% glycerin, 80% water/20% glycol, 50% water/40% glycerin/10% hexanediol and 60% water/30%glycerin/4% hexanediol/6% diacetone alcohol. Their properties are listed in Table 2. Glycerin and glycol influence the viscosity of the liquid. Hexanediol adjusts the surface tension and diacetone alcohol changes the polarity. Furthermore 3 different grades of High Speed Inkjet (HSI) ink have been tested, 2 inks of each grade. The first type is a dye ink, where the colourants are in molecular dispersion. The second one is a pigment ink, which uses dispersed pigments as colourants. The third ink is a latex pigment ink, which contains pigments for the colouring and latex particles to enhance the ink fixation on the surface of the paper. Every measurement has been performed with two colours of each ink-type: yellow and magenta.

**Table 2:** Testing liquids used in this study and their properties: viscosity, surface tension and polarity

Liquids	Viscosity [mPa s]	Surface tension [mN m^−1^]	d*D*	d*P*	d*H*
Water	1.004	72.4	15.5	16	42.3
80% water/20% glycerin	1.6	65	15.9	15.6	39.3
80% water/20% glycol	1.7	71	15.8	15	39.4
50% water/40% glycerin/10% hexanediol	6.2	27.3	16.36	13.2	33.74
60% water/30% glycerin/4% hexanediol/6% diacetone alcohol	3.45	33.2	16.1	13.75	42.9
Dye ink yellow	6.3	37	—	—	—
Dye ink magenta	6.3	35	—	—	—
Pigment ink yellow	6.3	36.9	15.8	13.8	35.6
Pigment ink magenta	6.3	37.4	15.8	13.8	35.6
Latex pigment ink yellow	5	32.8	15.8	13.8	35.6
Latex pigment ink magenta	5.3	33.4	15.8	13.8	35.6

### 2.3 Ultrasonic liquid penetration measurement (ULP)

**Fig. 1:**
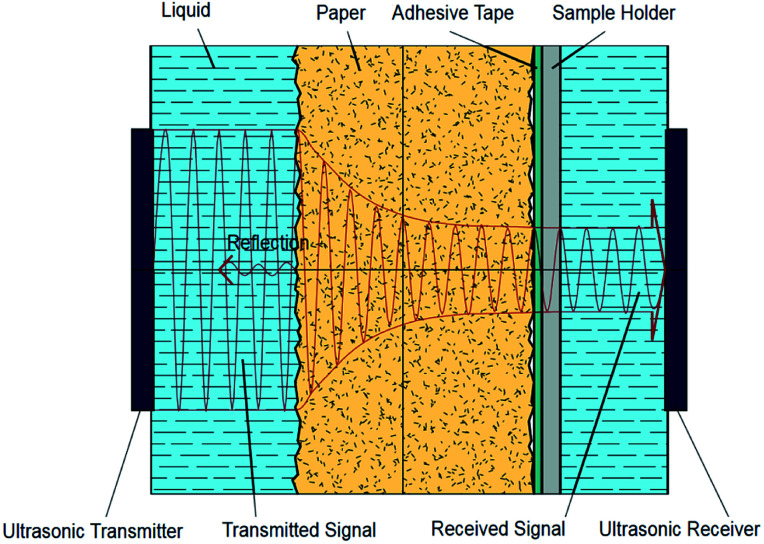
Measurement principle of the ultrasonic liquid penetration (ULP) measurement, drawing not to scale.^9^

The Emtec Penetration Dynamics Analyser 2.0 was used for all ultrasonic measurements. Measurement frequency was set to 2 MHz. The paper samples were cut to a rectangle of 7 cm × 5 cm and fastened to the sample holder with a two sided adhesive tape. In the measurement cell an ultrasonic emitter and an ultrasonic receiver are placed to the opposite of each other, shown in Fig. 1. When the sample is released into the testing cell filled with liquid, the transmitter instantly starts to send ultrasonic waves through the sample. The receiver measures the intensity of the ultrasonic signal. Sensor area is a circle with a diameter of 35 mm. The ultrasonic waves are reflected, scattered or absorbed during the process of liquid penetration, represented through the red lines in Fig. 1. As penetration of the liquid in the substrate proceeds, the receiver records the changes in the signal. The result is the ultrasound intensity over time.^9^

**Fig. 2:**
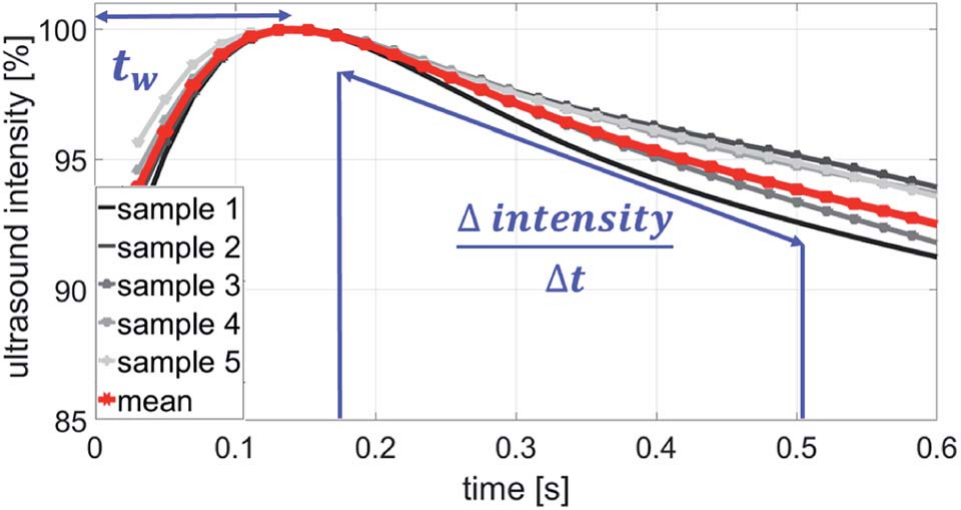
The ultrasonic measurement results show the change of ultrasound intensity over time. Time at the highest intensity *t*_w_ [s] is defined as wetting time of the liquid and the slope of the curve as the penetration speed 
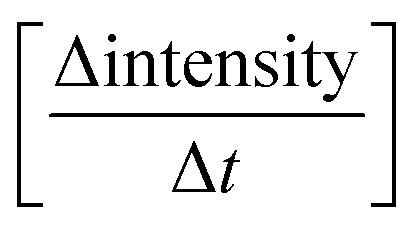
 of the liquid into the paper in [s^−1^].

A typical measurement result is shown in Fig. 2, it is from the AKD sized paper with one dye ink. The curves are results from 5 specimen of the same paper (grey) and their mean value (red). The wetting is represented as the wetting time, which is the time between liquid contact and the highest intensity (wetting time *t*_w_ in Fig. 2). The longer it takes to reach 100% intensity, the lower is the wetting. The penetration speed is calculated between the time at the highest intensity and approximately 200 ms for unsized papers and around 1 s for hydrophobized papers after this time. The faster the liquid penetrates into the paper, the higher is the change in ultrasound intensity and the steeper is the slope of the curve 
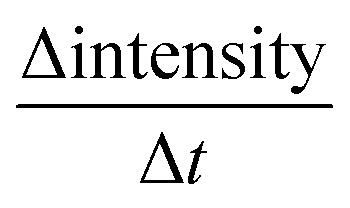
 (Fig. 2).

### 2.4 Contact angle measurement (CA)

**Fig. 3:**
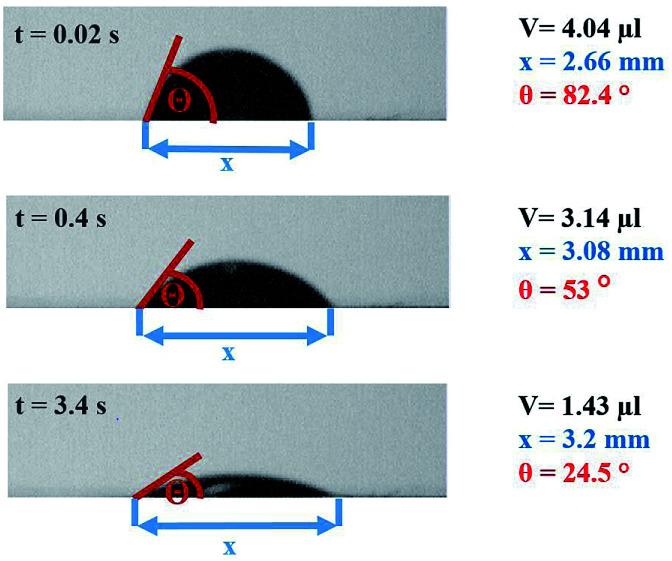
Drop at different times (0.02 s, 0.4 s, 3.4 s) and the resulting values for contact angle *θ*, width *x* and volume *V*.

**Fig. 4:**
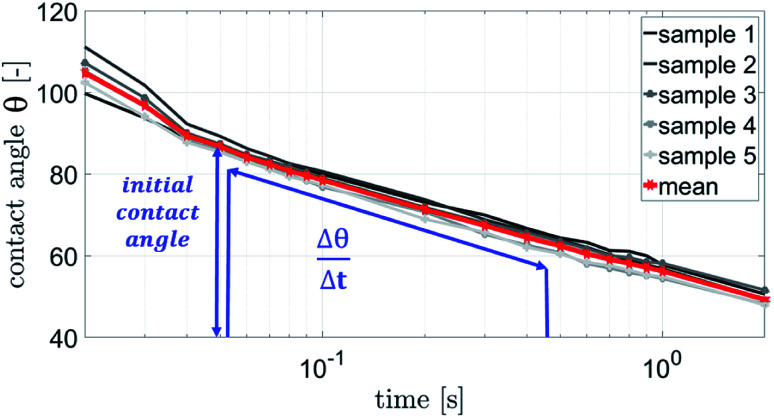
The contact angle plotted over time. Initial contact angle is measured after 0.05 s and the slope is measured between 0.05 s and 0.2 s. The initial contact angle is a measure for the liquid wetting of the surface. The slope 
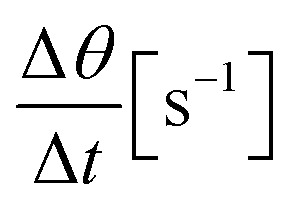
 is a parameter describing the combination of wetting and liquid penetration of the drop.

The contact angle measurements were performed using a Fibro DAT 1100 instrument.^30^ All measurements were carried out with 4 μl drop size. Once a drop is released onto the substrate's surface, the instrument starts taking pictures of the drop, as shown in Fig. 3. A tangent is fitted to the drop's outline at the contact point between paper and liquid using digital image analysis, and the contact angle (Fig. 3, red lines) is calculated. Also the width of the drop is measured and the drop volume on the surface is calculated. This is done for every picture the camera has taken. Three pictures of the same drop and their resulting values for contact angle, volume and width are shown in Fig. 3. The contact angle is plotted over time (Fig. 4). It is also possible to evaluate the total absorbed liquid volume per unit area (TLV/*A*). This is done by calculating the volume difference of a drop between different pictures and dividing it by the area the drop is covering. For calculating the volume of the liquid on the surface the drop shape is assumed to be spherical. The width of the drop is used to determine the area of the droplet on the substrate surface. The change of contact angle and contact area is caused by liquid flow into the substrate (penetration) and spreading of the drop on the surface (wetting). Evaporation can be neglected at this drop size.^31–33^ The initial contact angle is defined as the contact angle measured at 0.05 s after the drop has been put on the surface. A high contact angle is indicating bad wetting of the liquid on the surface. The higher the change of the contact angle over time, the faster is the liquid spreading and liquid penetration, and the steeper is the slope of the contact angle plotted over time as displayed in Fig. 4. The slope was calculated between 0.05 s and 0.2 s for the papers with treatments. The slope for the unsized & untreated paper was calculated between 0.05 s and 0.09 s, due to the fast penetration and wetting of the liquids.

### 2.5 Scanning absorptometer (SA)

**Fig. 5:**
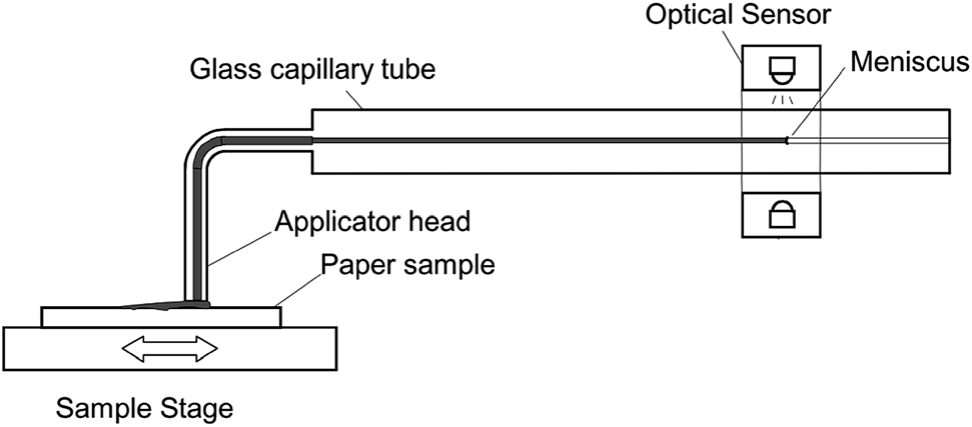
The scanning absorptometer set up. The applicator head on the paper sample is moved over the paper, ink is supplied with the liquid *via* a glass tube. The meniscus sensor follows the receding meniscus and computes the amount of liquid, which is absorbed in the paper. Adapted from Enomae *et al.*^12^

**Fig. 6:**
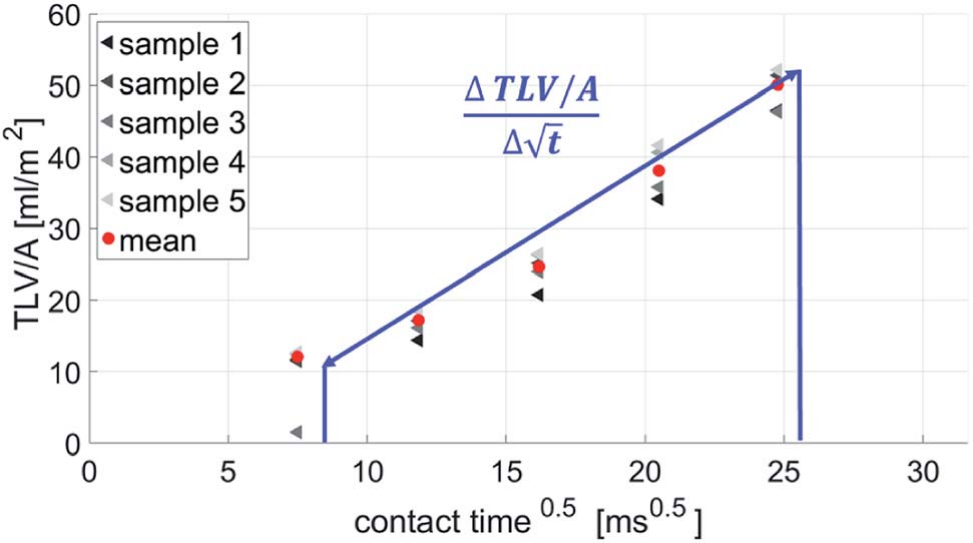
SA-measurement results show the absorbed liquid volume (total transferred liquid volume per unit area TLV/*A*) over time. The slope of the curve 
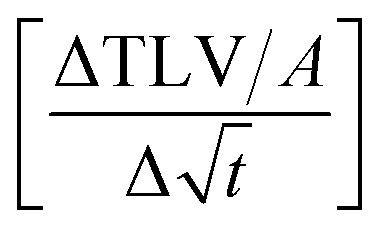
 represents the penetration speed in m s^−1/2^.

The scanning absorptometer measurement, performed with a KM500win Automatic Scanning Absorptometer instrument from KRK Kumagai (Japan), provides quantitative information about the liquid absorption as a function of time on time scales of 10 ms up to 10 s.^12,17^ During an SA measurement, liquid is supplied from a scanning head which moves along a spiral path on the paper. In Fig. 5 one can see the head on the paper sample surface, it is supplied with the liquid *via* a tube. The speed of the head moving over the paper surface is kept constant over a certain part of the track, then it accelerates stepwise to a higher speed which is then again kept constant. By increasing the speed the system measures the liquid penetration for different times of contact between the nozzle and the paper. The SA measures the total absorbed liquid volume per unit area. The penetration speed is represented by the slope 
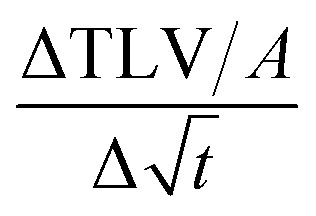
 of the curve. The steeper the slope, the higher the penetration speed (Fig. 6). The slope of this curve is calculated within the same time range as the slope of the ultrasonic liquid penetration measurement is calculated *i.e.* a contact time between approximately 0.031 ms and 0.200 ms after contact between liquid and paper was made. This is done for every single liquid/paper combination. Also a parameter indicating the wetting behavior is evaluated, it is the contact angle cos(*θ*)_LW_ calculated from the liquid penetration result as described in 2.6.

### 2.6 Contact angle calculated from scanning aabsorptiometry (SA) and the Lucas–Washburn equation

There are numerous theoretical studies on liquid flow into a porous system.^1,5,6,16,22,34,35^ Two models are often used: the Bosanquet model and the Lucas–Washburn (LW) equation. The LW approach is a quite simplified model system, assuming straight, circular capillaries with steady state liquid flow. It describes the flow regime where the driving forces, *i.e.* the pressure difference caused by the capillary forces of the liquid in the pore, are equal to the friction forces (the viscous forces).^34,36^ For penetration times below 1 ms at a pore diameters below 1 μm the LW-equations results differ from the measured values. Steady state flow is there not a valid assumption and inertial force needs to be considered for these conditions.^10,37,38^ The Bosanquet model is adding the inertial forces to the Lucas–Washburn equation.^39^ It has no restrictions in terms of time and pore size. However, Schoelkopf^10^ and Ridgway^37^ showed that the Lucas–Washburn is valid for our liquids and the pore system that we have in our papers, namely penetration times larger than 10 ms, and pore size diameters between 2.6 μm and 4.9 μm. Also earlier the Lucas–Washburn approach has successfully been used to describe the liquid flow into paper.^2,40^1
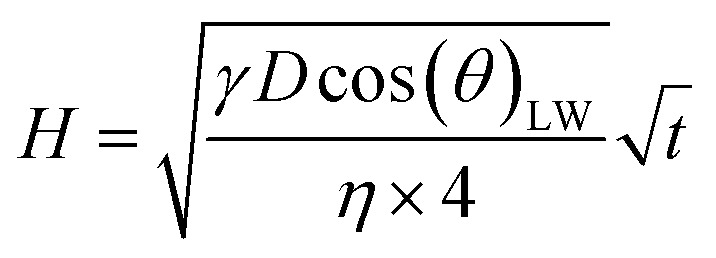


**Fig. 7:**
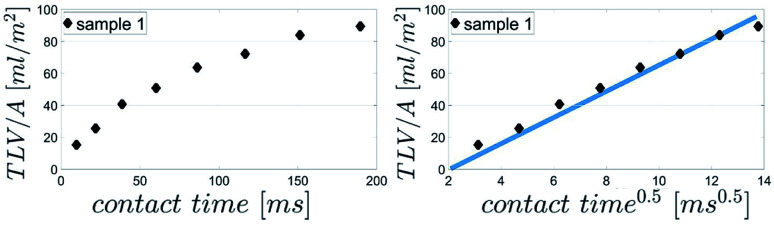
Comparison of SA results plotted over *t* (left side) and over 
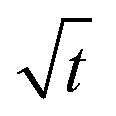
 (right side). The right diagram shows that *H* is proportional to 
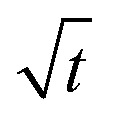
 as it is the case for Lucas–Washburn flow, eqn (1).

Eqn (1) gives the Lucas–Washburn equation, describing the liquid uptake into a porous media modeled by cylindrical capillaries. The penetration length *H* [m] is the distance traveled by the fluid in time *t* [s]. The parameters that influence *H* are the capillary diameter (pore diameter) *D* [m], the surface tension *γ* [N m^−1^] and the viscosity *η* [N s m^−2^] of the liquid and finally the contact angle *θ*_LW_ [-] between the liquid and the pore material. Eqn (1) shows that according to the LW equation the penetration depth H of the liquid into the substrate is directly proportional to 
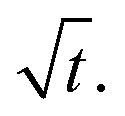
Fig. 7 shows the results for 1 SA measurement plotted over *t* on the left side. On the right side of Fig. 7 the same results are plotted over 
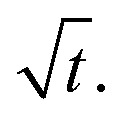
 A linear relation between *H* and 
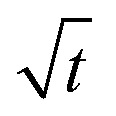
 can indeed be observed.

Please note that according to eqn (1) we can calculate the contact angle *θ*_LW_ when we know all the other parameters *H*, *D*, *γ* and *η*. This is exactly the idea of our analysis, as a measure for surface wetting we are calculating the contact angle according to the Lucas–Washburn equation *θ*_LW_ from the scanning absorptometer measurement results.

Eqn (2) defines the volume uptake of a porous medium consisting of several parallel capillaries with the pore diameter *D* [m]. The number of pores within an area *A* is *N*_p,A_ = *N*/*A* [m^−2^] and each capillary has the volume 
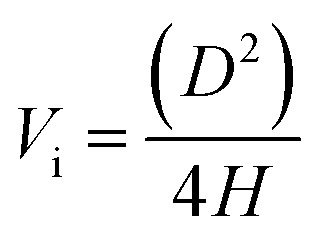
 [m^3^]. The number of capillaries (pores) *N* multiplied with the volume *V*_i_ [m^3^] of each capillary is the total volume uptake *V*:^17,41^2
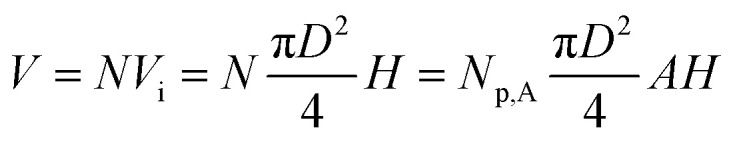


Defining porosity *ε* as 
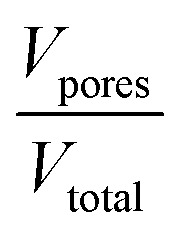
 we find3
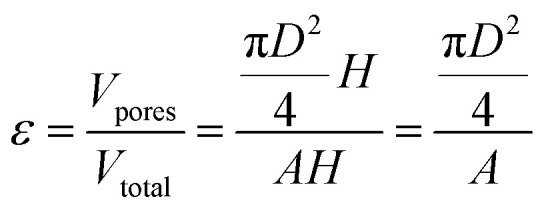


It follows that the total absorbed liquid volume per unit area 
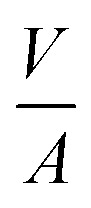
 is equal to the porosity multiplied with the penetration length:4
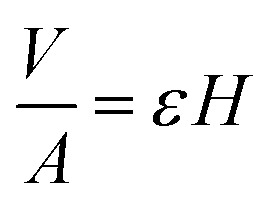


The travelled distance of the liquid flow into the porous media *H* is described by the LW equation. Therefore the term 
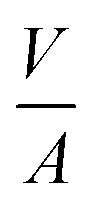
 is determined by substituting *H*, eqn (1), into eqn (4). Rearranging the resulting expression leads to5
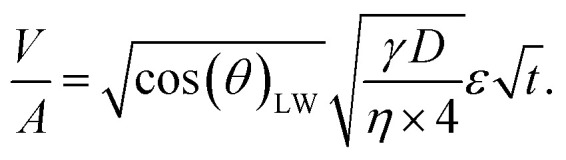


Eqn (5) can be re-written as a linear equation *y* = *kx* with 
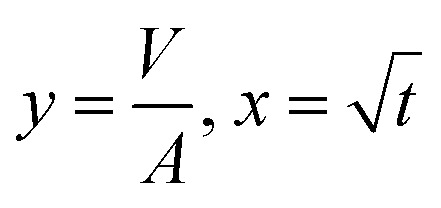
 and the slope as 
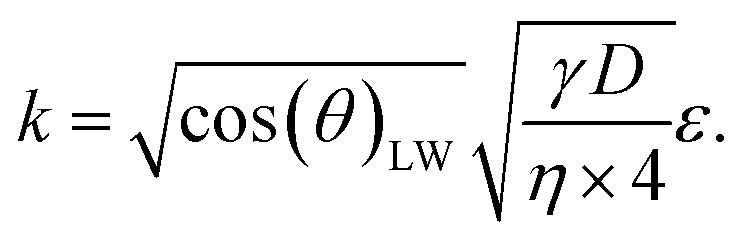


**Fig. 8:**
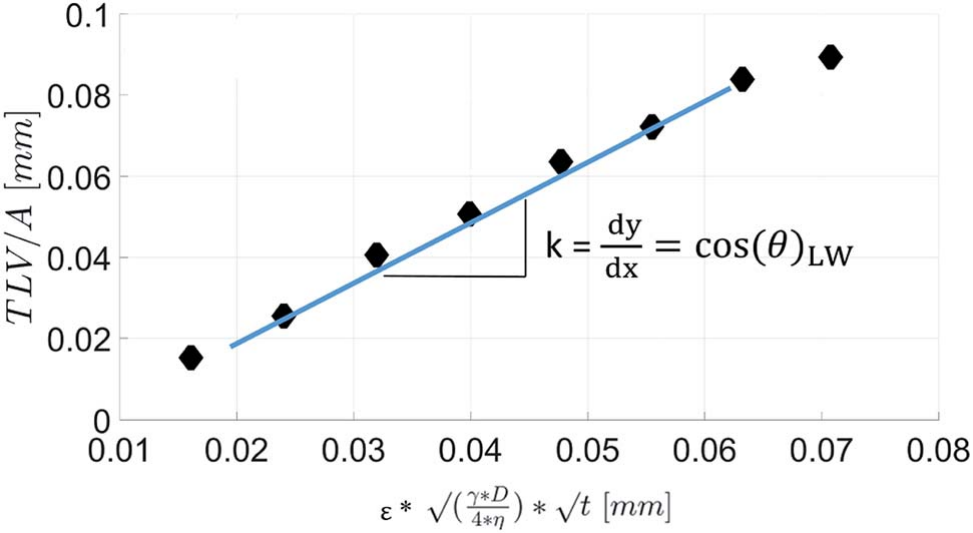
The scanning absorptopmetry (SA) results are plotted over 
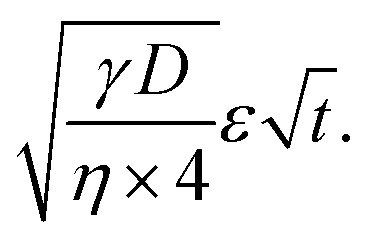
 The result is a straight line, indicating that the assumption of Lucas–Washburn flow is justified. The slope *k* of the line is equivalent to 
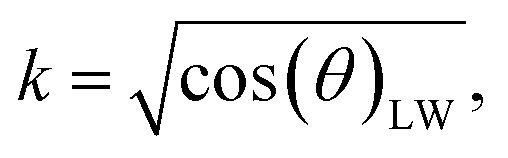
*i.e.* the square root of the contact angle.

By plotting the result of the SA measurement 
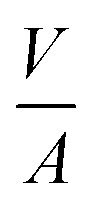
 on the *y*-axis and 
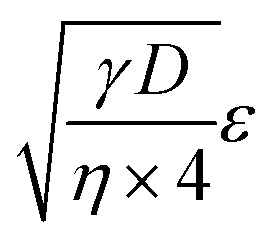
 on the *x*-axis we thus find 
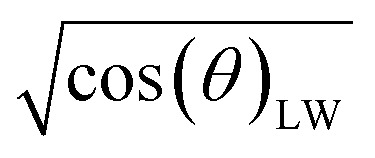
 as the slope in the resulting diagram, compare Fig. 8. The values for *t* are taken from the SA measurements. The surface tension *γ* [*N* m^−1^] and the viscosity *η* [N s m^−2^] of the liquid have been measured for each fluid, Table 2. The parameters capillary diameter *D* [m] and the porosity *ε* [-] for the pore system have been measured for each paper, Table 1. Evaluating the slope of each SA penetration measurement it is possible to obtain a value for wetting, cos(*θ*)_LW_, for each combination of liquid and paper from the SA. This value cos(*θ*)_LW_ can be interpreted as the paper-liquid contact angle measured from the liquid penetration into the paper, under the assumption of Lucas–Washburn flow.

## 3 Results

### 3.1 Penetration speed

**Fig. 9:**
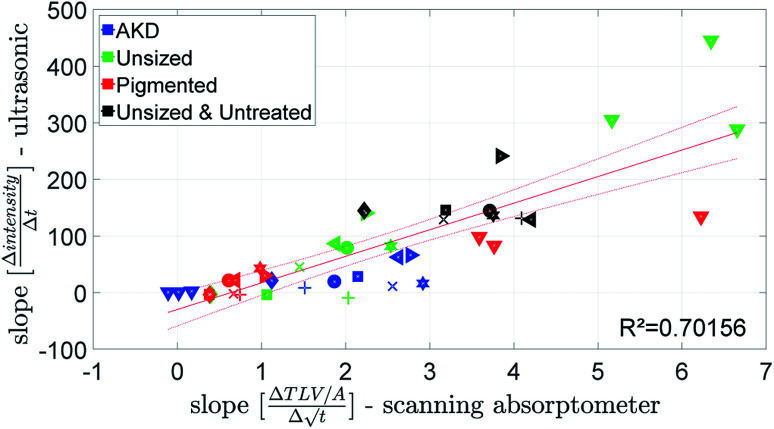
Penetration speed from the ultrasonic measurement and the scanning absorptometer. For detailed description of the symbols refer to the ESI.†

Fig. 9 shows the penetration speed measured by the ultrasonic measurement (ULP) plotted against the penetration speed detected by the scanning absorptometer (SA). The absorption of the model liquids water, water/glycerin and water/glycol into the untreated & unsized paper was too fast and could not be detected by the scanning absorptometer. Both measurements show for the unsized papers and the liquids without hexanediol the fastest liquid uptake, followed by the liquids with hexanediol and the inks. Looking at the results for the AKD sized paper the model fluids which have a water content of 80% and 100% penetrate really slowly into the paper which is found by both techniques (Fig. 9). So, despite the totally different measurement principle, ULP and SA are delivering fairly similar results.

**Fig. 10:**
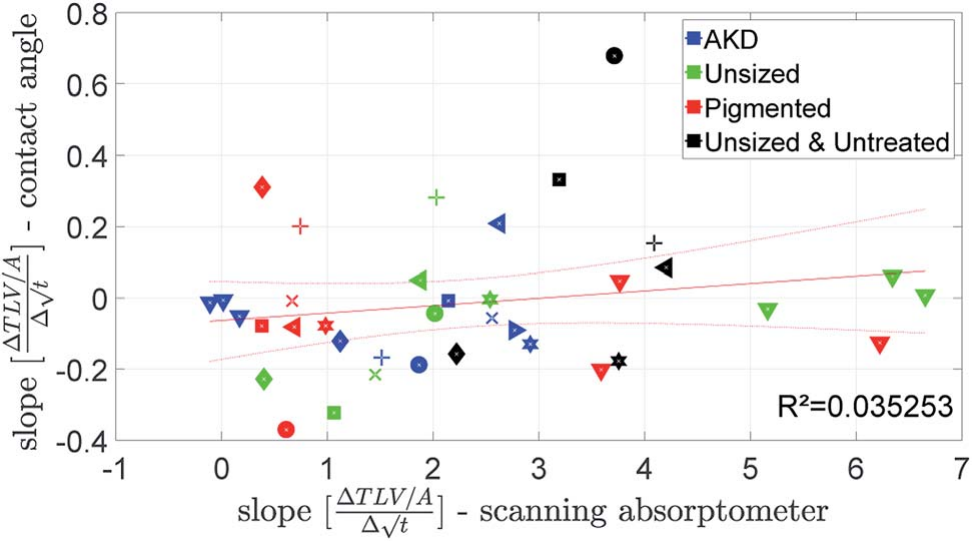
Penetration speed from contact angle measurement and scanning absorptometer. Symbols are described in ESI.†

**Fig. 11:**
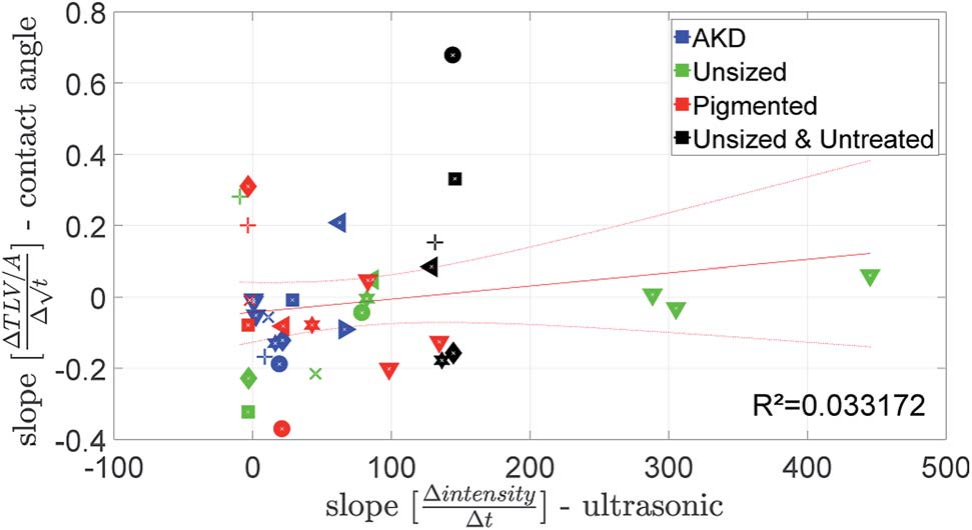
Penetration speed from ultrasonic measurement and contact angle measurement. Symbols are described in ESI.†

The penetration speed calculated from the contact angle measurement (CA) is neither correlating well to the SA, nor to the ULP data (Fig. 10 and 11). We believe the main reason is that the supplied liquid volume is comparably small (4 μl) and for the contact angle device the contact area where penetration takes place is influenced by drop spreading. In the literature^42^ it has been shown that penetration into paper is considerably slower for a limited supply of liquid than for an unlimited supply. We thus believe that the differences in the measurement results represent the different penetration behavior between large amounts of liquid and small drops.

### 3.2 Wetting

The parameter for wetting from the ULP measurement is the wetting time *t*_w_, see Fig. 2. The wetting parameter from the contact angle measurement is cos(*θ*)_50 ms_ the cosine of the initial contact angle measured after 50 ms, Fig. 4.

**Fig. 12:**
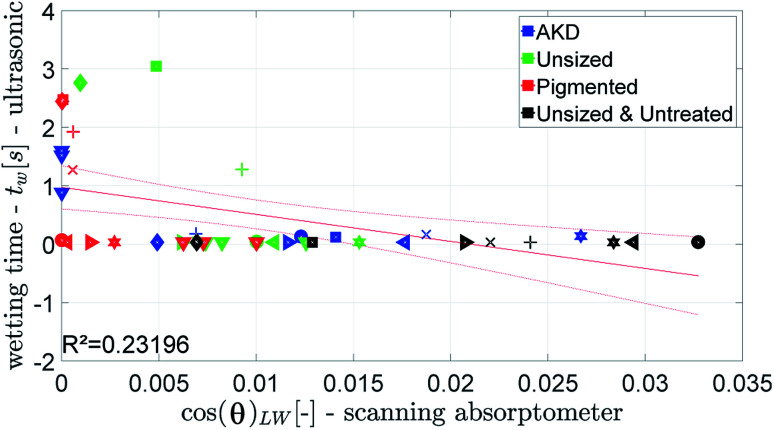
Ultrasonic wetting time compared to the value for cos(*θ*)_LW_ calculated from the scanning absorptometer data. Symbols are described in ESI.†

**Fig. 13:**
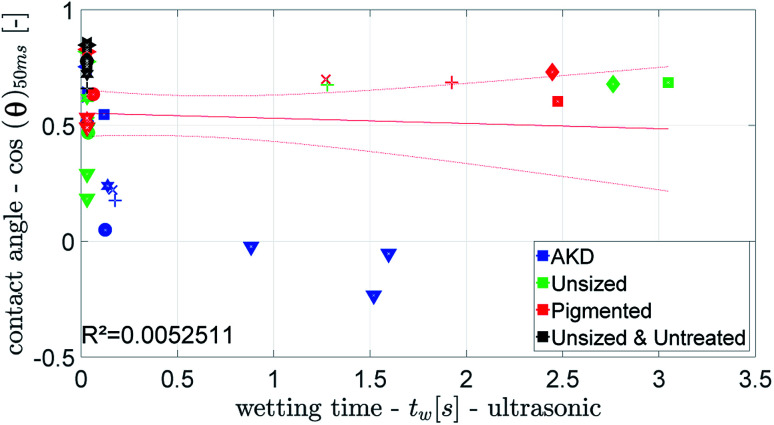
Ultrasonic wetting time compared to the wetting parameter from the CA. Symbols are described in ESI.†

Fig. 12 and 13 show that the ultrasonic measurement is not able to capture the wetting of unsized papers. For most results the measured wetting time is zero, it cannot be determined because it takes place too fast to be detected by the ULP instrument. Also for the papers with lower wetting (AKD and pigmented paper) no correlation between ULP and the other instruments can be found. We can thus conclude that the ULP instrument is not suitable for capturing the wetting behavior of these liquids on paper.

**Fig. 14:**
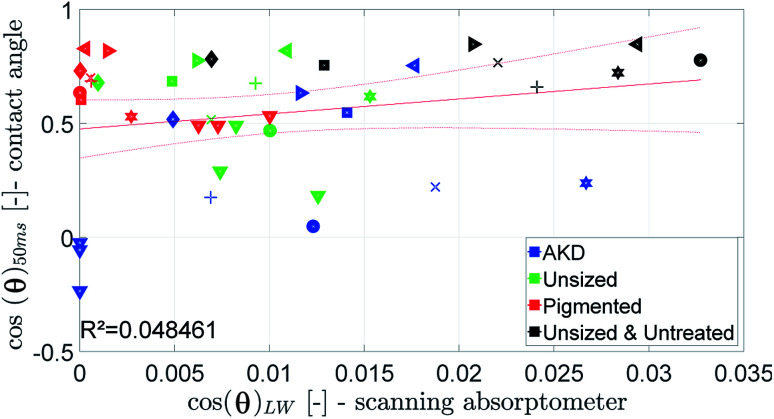
Contact angle measured by the contact angle measurement at 50 ms compared to the value for cos(*θ*)_LW_. Symbols are described in ESI.†

The parameter for the spreading of the liquid measured by the SA is cos(*θ*)_LW_, the contact angle calculated from the liquid penetration into the paper according to Section 2.6. It is found that the cosine of the initial contact angle directly measured by the CA instrument does not correlate well to cos(*θ*)_LW_ calculated from the SA liquid penetration measurement (Fig. 14). It seems that the contact angle can not be calculated correctly using the Lucas–Washburn model. That comes somewhat surprising because the relationship between time and absorbed liquid volume was shown to have a 
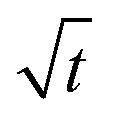
 proportionality, like predicted by the Lucas–Washburn equation, compare Fig. 7. Also others have found Lucas–Washburn behavior for liquid penetration in paper.^2,40^ A likely reason for the deviating results is the gross simplification of the pore system as a bundle of circular capillaries with one constant diameter. The pore system in paper has a complex geometry and a wide distribution of pore sizes.^28^ It seems that while overall the penetration is following a Lucas–Washburn type time dependency, for our substrate we need a more complex pore model than the one in the Lucas–Washburn equation to successfully calculate wetting from the liquid penetration.

In conclusion we found that ULP and SA did not deliver results for the wetting behaviour that can be compared to standard contact angle measurements.

### 3.3 Combined measurement parameter for penetration speed and wetting

**Fig. 15:**
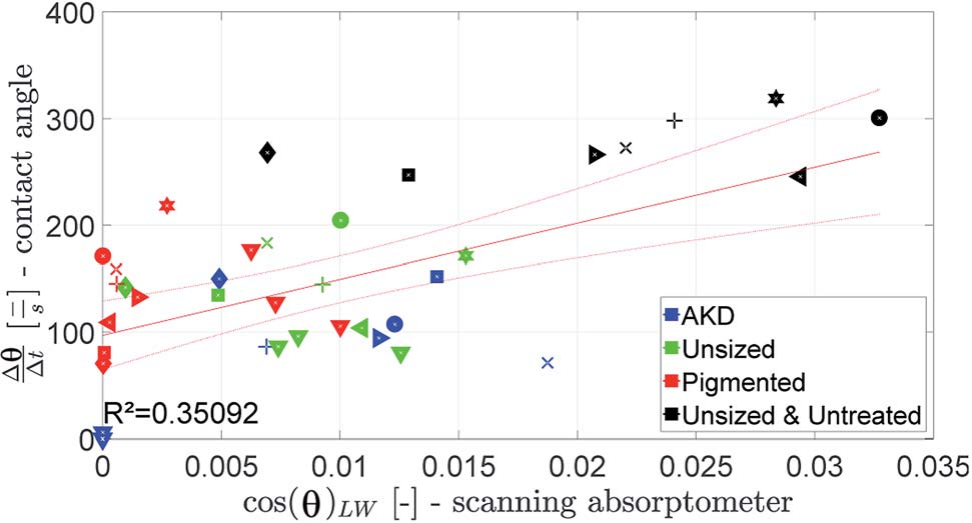
The slope of the contact angle curve compared to the value for cos(*θ*)_LW_ calculated from the scanning absorptometer. Symbols are described in ESI.†

A combined parameter for liquid penetration and wetting is the slope of the contact angle curve. It reflects the wetting and the liquid penetration of the drop, which takes place during the time range of 0.05–0.2 s (0.05–0.09 s for fast absorbing liquids), compare Fig. 4. This parameter fits better (*R*^2^ = 0.35) to the cos(*θ*)_LW_ value calculated from the scanning absorptometer (Fig. 15) results. The correlation indicates that cos(*θ*)_LW_ is in fact also a combined parameter which is reflecting both, the wetting on and the penetration of the fluid into the substrate. Considering that cos(*θ*)_LW_ is the contact angle which has been calculated from the liquid penetration measurement, this interpretation seems plausible. The correlation between the measurements is low (*R*^2^ = 0.35), still they somehow seem to capture similar aspects of liquid–paper interaction.

## 4 Conclusions

Ultrasonic liquid penetration measurement, contact angle measurement and scanning absorptometer are all measuring liquid penetration and surface wetting, using totally different measurement principles. In this study parameters for surface wetting and liquid penetration speed have been defined for each measurement technique and the results of the measurements have been compared. For our studies we have developed and used model liquids to independently control surface tension, viscosity and polarity of the testing fluids.

The penetration speed measurement from ULP and scanning absorptiometry showed similar results, indicating that both measurements, despite their entirely different measurement principle, are capturing the liquid penetration speed into the paper. In contrast to that liquid penetration speed measured from individual drops on the surface of the substrate gave results that differed from the other two techniques. We think that the reason might be that for the individual drops the liquid absorption is influenced by the drop spreading on the surface, while for the other two analysis techniques the contact area where penetration takes place is not influenced by wetting. We thus conclude that the penetration of small drops thus exhibits a fundamentally different penetration behavior than large amounts of liquid applied to the substrate.

For the surface wetting behaviour all three measurements techniques gave different results. We thus conclude that the well established contact angle measurement remains the most useful approach, and that ULP and SA did not provide meaningful results here.

Finally we have defined a combined parameter describing wetting and liquid penetration, it is the change in contact angle of a drop over time. This parameter is driven by both, spreading of the drop and penetration of the liquid into the substrate. A moderate correlation was found to a parameter derived from scanning absorptiometry, namely the contact angle calculated from liquid penetration using the Lucas–Washburn equation. It seems that both parameters are describing a combination of wetting and liquid penetration.

### 4.1 Implications for measurement of contact angle and liquid penetration on thin porous materials

The results have demonstrated that for measurement of wetting and liquid penetration on thin, porous materials the timescale and the size scale of the measurement is highly relevant. The time scale for all measurements was controlled it was 200 ms for fast penetration and 1.5 s for slow penetration. The size scale was differing between the measurements, excess amounts of liquids are being applied in the ultrasound measurement and for scanning absorption measurements, 4 μl drops for the contact angle measurement. Accordingly the absorption measurements of ULP and SA agree but the CA absorption is not correlated to the others. For evaluation of surface wetting only contact angle measurements are providing reasonable results. However on absorbing materials the contact angle is always a combined measurement of wetting and liquid penetration, compare Fig. 3 and 4. Thus it is particularly relevant to take drop size and measurement time into account.

The interpretation of wetting and penetration measurements on thin porous materials always has to consider the time scale of penetration and the size scale of the drop/substrate system. In the case of ink jet printing these are picoliter drops penetrating within milliseconds. In the case of other absorbing media it might be liters taken up within days. The difference in the results between the methods evaluated in this work are demonstrating the importance to carefully select the measurement method for wetting (contact angle) and liquid penetration in such a way that it reflects the time scale and size scale of the industrial application to be analyzed.

## Conflicts of interest

There are no conflicts of interest.

## Supplementary Material

RA-008-C8RA01434E-s001
