# Supplementary material for: Short timescale wetting and penetration on porous sheets measured with ultrasound, direct absorption and contact angle
Source: RSC Adv. 2018 Apr 4;8(23):12861–9. doi: 10.1039/c8ra01434e (PMC9079626; doi:10.1039/c8ra01434e)
Supplement: RA-008-C8RA01434E-s001 [file RA-008-C8RA01434E-s001.pdf]

AKD - Water
AKD - 80%Water/20%Glycerin
AKD - 80%Water/20%Glycol
AKD - 50%Water/40%Glycerin/10%HD
AKD - 60%Water/30%Glycerin/4%Hexanediol/6%Diacetonalcohol
AKD - dye ink yellow
AKD - dye ink magenta
AKD - pigment ink yellow
AKD - pigment ink magenta
AKD - latex pigment ink yellow
AKD - latex pigment ink magenta
Unsized - Water
Unsized - 80%Water/20%Glycerin
Unsized - 80%Water/20%Glycol
Unsized - 50%Water/40%Glycerin/10%Hexanediol
Unsized - 60%Water/30%Glycerin/4%Hexanediol/6%Diacetonalcohol
Unsized - dye ink yellow
Unsized - dye ink magenta
Unsized - pigment ink yellow
Unsized - pigment ink magenta
Unsized - latex pigment ink yellow
Unsized - latex pigment ink magenta
Pigmented - Water
Pigmented - 80%Water/20%Glycerin
Pigmented - 80%Water/20%Glycol
Pigmented - 50%Water/40%Glycerin/10%Hexanediol
Pigmented - 60%Water/30%Glycerin/4%Hexanediol/6%Diacetonalcohol
Pigmented - dye ink yellow
Pigmented - dye ink magenta
Pigmented - pigment ink yellow
Pigmented - pigment ink magenta
Pigmented - latex pigment ink yellow
Pigmented - latex pigment ink magenta
Unsized & Untreated - 50%Water/40%Glycerin/10%Hexandiol
Unsized & Untreated - 60%Water/30%Glycerin/4%Hexanediol/6%Diacetonalcohol
Unsized & Untreated - dye ink yellow
Unsized & Untreated - dye ink magenta
Unsized & Untreated - pigment ink yellow
Unsized & Untreated - pigment ink magenta
Unsized & Untreated - latex pigment ink yellow
Unsized & Untreated - latex pigment ink magenta
